# CO_2_ laser versus cold steel margin analysis following endoscopic excision of glottic cancer

**DOI:** 10.1186/1916-0216-43-6

**Published:** 2014-02-06

**Authors:** Fawaz M Makki, Matthew H Rigby, Martin Bullock, Timothy Brown, Robert D Hart, Jonathan Trites, Michael L Hinni, S Mark Taylor

**Affiliations:** 1 Department of Surgery, Division of Otolaryngology Head & Neck Surgery, Dalhousie University, Halifax, Nova Scotia, Canada; 2 Department of Pathology, Dalhousie University, Halifax, Nova Scotia, Canada; 3 Department of Otolaryngology-Head and Neck Surgery, Mayo Clinic, Phoenix, AZ, USA; 4 Suite 3052, Dickson Bldg., 5820 University Avenue, Halifax, NS B3H 1 V9, Canada

**Keywords:** Glottic cancer, CO_2_ Laser surgery, Tumor margin

## Abstract

**Objective:**

To compare the suitability of CO_2_ laser with steel instruments for margin excision in transoral laser microsurgery.

**Methods:**

Prospective randomized blinded study. Patients with glottic cancer undergoing laser resection were randomized to margin excision by either steel instruments or CO_2_ laser. Margins were analyzed for size, interpretability and degree of artifact by a pathologist who was blinded to technique.

**Results:**

45 patients were enrolled in the study with 226 total margins taken. 39 margins taken by laser had marked artifact and 0 were uninterpretable. 20 margins taken by steel instruments had marked artifact, and 2 were uninterpretable. Controlling for margin size, the laser technique was associated with increasing degrees of margin artifact (p = 0.210), but there was no difference in crude rates of uninterpretability (p = 0.24).

**Conclusion:**

Laser margin excision is associated with a greater degree of artifact than steel instrument excision, but was not associated with higher rate of uninterpretability.

## Introduction

In 1972, Strong and Jako were the first to report the use of transoral laser microsurgery (TLM) in the treatment of glottic cancer [1]. Since the early 1990s, the indications for TLM have expanded to include all tumor categories of the upper aero-digestive tract [2-5]. Alongside with radiation therapy, TLM has become one of the primary modalities in the treatment of early glottic cancer [6-10]. In TLM, as with the rest of head and neck cancer surgery, local control is maximized by complete excision with adequate margins. Positive margin status in glottic cancer has been associated with increased risk of local recurrence and poorer prognosis [11-14].

Despite the importance of achieving clear margins in head and neck cancer surgery, there is no consensus on how wide a surgical margin is needed to be defined as “clear” [15-17]. In glottic cancer over excision of normal tissue can unnecessarily impair post-treatment vocal function. Several authors have suggested that in glottic cancer a margin can be considered free if the distance to the disease is at least 1 mm [15,16,18,19].

The degree of artifact in margin specimens is another important factor that can affect the interpretation of a margin. An artifact on histological examination refers to an alteration of tissue or cellular structures that resulted from an external factor [20]. These artifacts can be attributed to either trauma from surgical instruments, or the various stages of histopathology slide preparation (fixation, processing, embedding, sectioning, or staining of tissues sections) [20,21]. Artifacts from surgical excision technique can take many forms including crush injury, haemorrhage, splitting or fragmentation [20,22-26]. In addition to these artifacts, the use of electrocautery and laser can cause tissue fulguration from the thermal damage [27]. As artifact increases, the interpretability of margins decreases. Uninterpretability or misinterpretation of a margin due to artifact can have significant effects on both downstream treatment pathways and prognosis for a patient.

Several modifications to the application of carbon dioxide (CO_2_) in TLM have been made to minimize the thermal damage. Many of these modifications have been studied in procedures involving benign lesions of the vocal cords. Several studies have showed that the use pulsed CO_2_ laser is superior and has better wound healing when compared to continuous wave CO_2_ laser as it allows tissue cutting and hemostasis while limiting thermal damage to surrounding tissues [28-31]. The two most common pulsed modes in TLM using CO_2_ laser are ultrapulse and superpulse (pulse duration < 1 millisecond), both perform a precise cut while producing less damage to the surrounding tissue [31]. Cutaneous and mucosal incisions made by CO_2_ laser in the continuous wave mode were compared to cold steel resulting in significant thermal damage to surrounding tissue and delayed wound healing [32-36]. However, when used in the pulsed mode for either benign vocal cord lesions or cutaneous incisions both vocal outcomes and wound healing were found to be comparable [34,37,38].

Removal of adequate glottic resection margins after TLM can be technically challenging, and margins must be taken precisely to balance the need for sufficient tissue to analyze while maximizing the preservation of normal tissue. Steel instruments have traditionally been used to take margins due to concerns regarding interpretation of margins with laser artifact. Unfortunately, margin resection with steel instruments does not have the hemostatic benefits of CO_2_ or the stabilized cutting of the micromanipulator. As the pulsed setting of the laser significantly reduces the amount of thermal damage and artifact, margins at our center have routinely been taken by ultrapulsed laser to achieve these benefits.

The main objective of this study is to compare the degree of artifact and the rate of uninterpretability for glottic cancer margin specimens when excised using either CO_2_ laser on ultrapulse mode or steel phonomicrosurgical instruments. Our hypothesis is that margin excision using CO_2_ laser on ultrapulse mode can be as interpretable as using steel instruments.

## Methods

This is a blinded randomized trial approved by the Capital Health research ethics board. Patients were enrolled from multidisciplinary head and neck oncology clinic. All participants provided informed consent prior to inclusion in the study.

### Subjects

**Figure 1:**
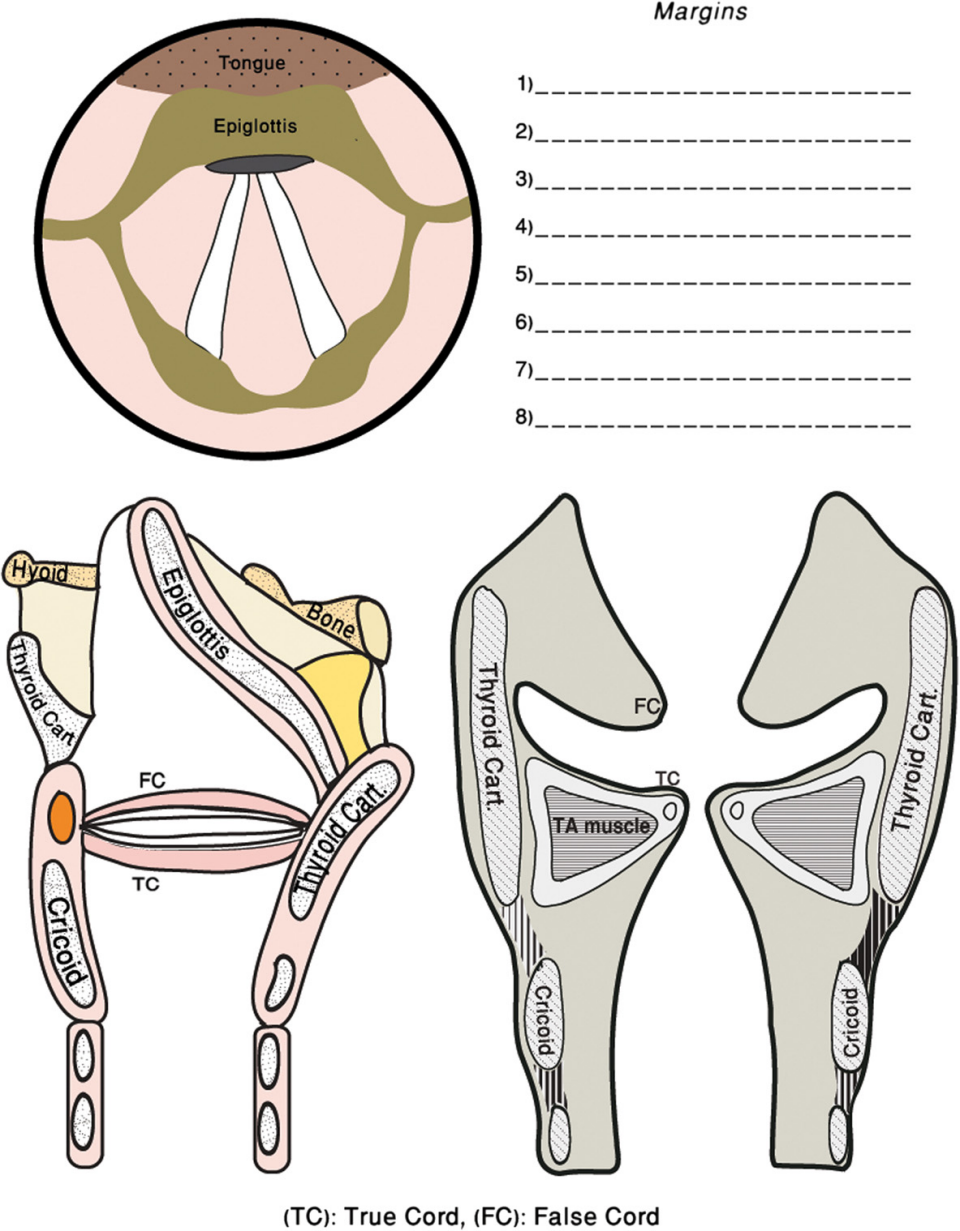
Laryngeal cancer template.

All patients ≥ 18 years old undergoing TLM as primary modality for T1 or T2 glottic cancer from January 2010 to Dec 2011 were eligible for enrolment in the study. Patients with locally advanced glottic cancer (T3 & T4), undergoing TLM for recurrence, or salvage post-radiation therapy were excluded. Patients were randomized to margin excision with either CO_2_ laser or steel instruments using a computer generated random list. The patient and the pathologist were both blinded as to what technique was used for margin acquisition. Specimens were all submitted to the pathologist with a laryngeal template (Figure 1) indicating the site of excision without the mention of technique used. Only one pathologist (MB) and one surgeon (SMT) was involved in the study.

### Surgical procedure

Patients were placed under general anaesthesia and intubated using laser resistant endotracheal tube. Laser safety measures were implemented to minimize the risk of complications from the laser surgery. Patients were placed in suspension laryngoscopy and an operative microscope with CO_2_ laser micromanipulator was used for glottic cancer excision. Carbon dioxide laser settings were 2-4 watts in ultrapulse mode. Depending on size, the tumours were removed either *en bloc* or via a tumour splitting technique. After primary tumor excision, margins were taken from the surgical site using either steel phonomicrosurgical instruments or CO_2_ laser at 2 watts based on our randomization list. The same phonomicrosurgical instruments were used to retract specimens for both cutting techniques. The locations of margins were diagrammed on the laryngeal template to assist the pathologist in their orientation.

### Histopathology examination

**Figure 2:**
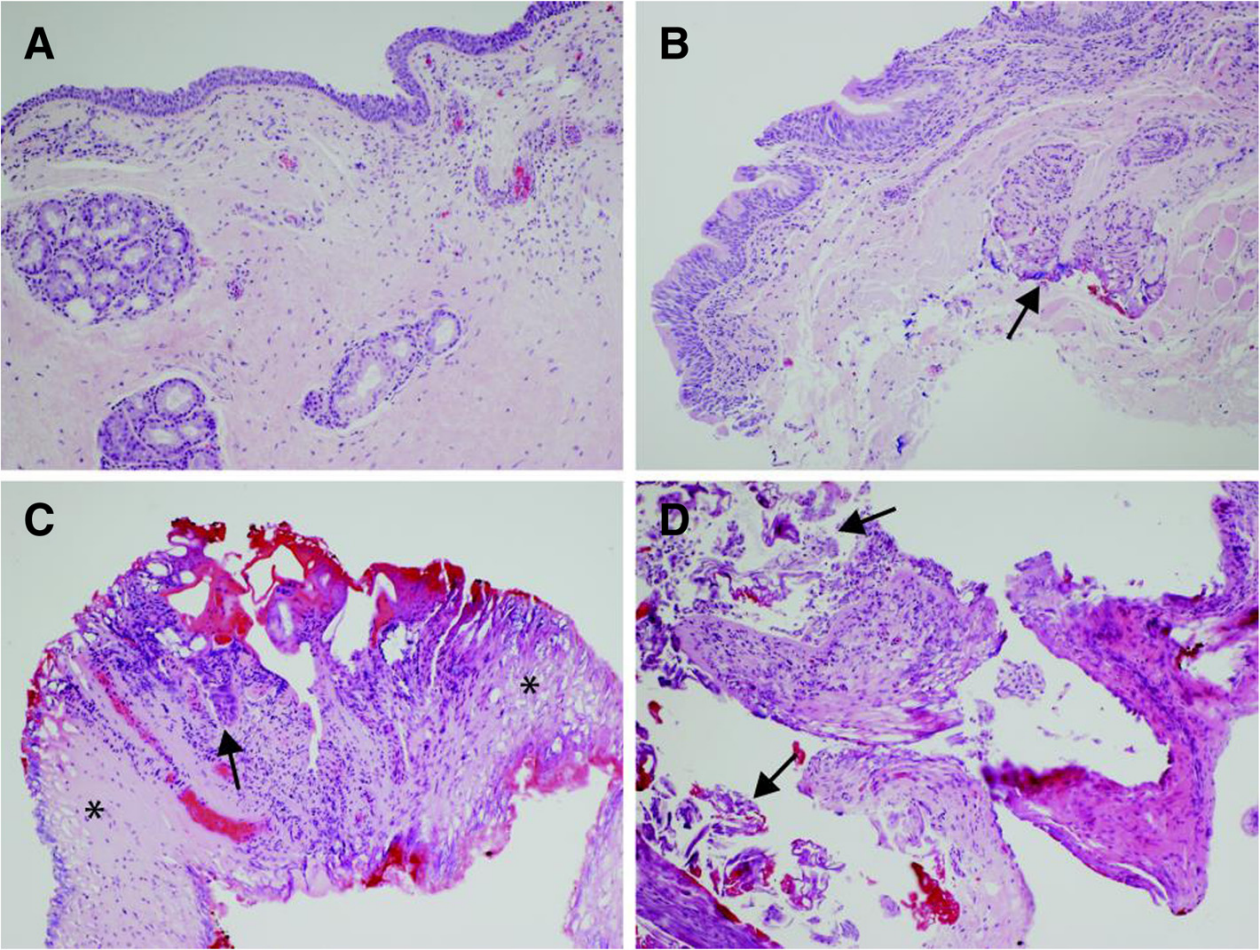
**Examples of histological grading system for artifact. *A)****None:* Well preserved surface epithelium, seromucinous glands and stroma. ***B)****Minor:* Well preserved surface epithelium. Seromucinous glands show some indistinct hyperchromatic areas (arrow). ***C)****Marked:* Surface epithelium is partially denuded with remainder showing severe artifact (arrow). Lamina propria is relatively preserved and can be assessed for invasive carcinoma (asterisk). ***D)****Uninterpretable:* Fragmented margin tissue with detached surface epithelium (arrow) and indistinct fragments of lamina propria.

All margin specimens were sent to the same pathologist who was blinded to margin excision technique. Prior to the study, a trial of margin excision using both techniques was performed and reviewed with the pathologist to assess for obvious signs of thermal artifact. On histological analysis there weren’t any obvious difference between the two techniques, which allowed for blinding of the pathologist to technique. Histopathology slides were prepared as per standard protocol. The specimens were fixed in 10% formalin immediately following excision, paraffin-embedded and then 5-micron sections were stained with hematoxylin and eosin. For each margin, the pathologist was provided with three levels through the tissue at approximately 40-micron intervals. All margin specimens were examined for their size (following formalin fixation but prior to embedding in paraffin), malignancy/dysplasia status, and degree of artifact. For the study artifact was defined as an alteration of tissue or cellular structures that resulted from an external factor. The degree of artifact was divided into: none, minor, marked, and uninterpretable as defined in Table 1 and shown in Figure 2.

**Table 1:** Classification system for the degree of artifact

**Degree of artifact**	**Definition**
**None**	No Artifact
**Minor**	Minor degree of artifact Still interpretable for both malignancy & dysplasia with accuracy
**Marked**	Greater degree of artifact, causing difficult interpretation of specimen Could interpret for margin malignancy status but not for the presence or degree of dysplasia with accuracy
**Uninterpretable**	No opinion on the presence or absence of dysplasia or malignancy could be given

### Data collection & statistical analysis

Data collected included demographic information, procedural details (margin excision technique, number of margin specimens, and location of each margin), and pathological description (degree of differentiation, size of margin specimens, margin status for dysplasia and invasive carcinoma, degree of dysplasia, grade of carcinoma, and degree of artifact).

Statistical analysis was performed using Stata v11.2 (StataCorp, Texas). The sample size was calculated to have 0.8 power to detect a difference in proportions of 0.1 in the crude rates of uninterpretability between techniques with a two-tailed α of 0.05. A pre-planned analysis was used to determine the relationship between margin interpretability and harvest technique. The study included two primary endpoints. These were set as the relationship between harvest technique and uninterpretable margins, and the relationship between harvest technique and marked artifact or uninterpretable margins. Two-tailed Fisher exact tests were used for this analysis. For all tests, significance was set at an α < 0.05 level. A descriptive analysis was performed for demographic variables. Analyses of variables between assigned groups were performed using Fisher exact tests for categoric variables, and T-test for continuous variables.

The secondary endpoints were to the relationship between harvest technique and margin size and the relationship between harvest techniques and marked artifact/uninterpretable margins independent of margin size. This was performed to assess the potential role of vaporization and destruction of tissue caused by laser excision in the interpretability of margins. A t-test was used to determine the relationship between harvest technique and margin size. A logistic regression model, controlling for margin size, was used to analyse the relationship between harvest technique and presence of either marked artifact or uninterpretable margins.

## Results

The study cohort was composed of 45 patients with 23 randomized to have margins taken by CO_2_ laser and 22 randomized to have margins taken with steel phonomicrosurgical instruments. See Table 2 for patient demographics. Overall, there were 226 margins taken, 115 margins taken with CO_2_ laser and 111 taken with steel phonomicrosurgical instruments (Table 3).

**Table 2:** Demographics and diagnosis

**Variable**	**Laser**	**Steel**	**Total**	**p-value**
**Age**				0.548
*Mean*	65.2	67.4	66.3	
*Range*	-	-	34-87	
*SD*	11.5	12.7	12.0	
**Gender**				1.0
*Male*	20	19	39	
*Female*	3	3	6	
**Diagnosis**				0.463
**a) Premalignant**				
*Moderate dysplasia*	0	2	2	
*Severe dysplasia/CIS*	6	3	9	
**b) Invasive SCC**				
*Well differentiated*	2	2	4	
*Moderately differentiated*	12	14	26	
*Poorly differentiated*	3	1	4	

**Table 3:** Margins excision technique and their degree of artifact

**Degree of artifact**	**Laser**	**Steel**	**Total**
***None*** *(% of technique)*	1 (0.9)	6 (5.4)	7 (3.1)
***Minor*** *(% of technique)*	75 (65.2)	83 (74.8)	158 (69.9)
***Marked*** *(% of technique)*	39 (33.9)	20 (18.0)	59 (26.1)
***Uninterpretable*** *(% of technique)*	0 (0)	2 (1.8)	2 (0.9)

### Primary analysis

There were no margins taken by laser (n=115) that were uninterpretable (0%), and 2 margins taken by steel instruments (n=111) that were uninterpretable (1.8%). The difference between these was not significant (p = 0.24). There were 39 (33.9%) margins taken by laser that had marked artifact, and 22 (19.8%) margins taken by steel instruments that had either marked artifact [20] or were uninterpretable [2]. The difference between these was statistically significant (p = 0.024) with margins taken by laser having a relative risk of 1.7 (95% CI 1.0-2.7) for having either marked artifact being uninterpretable.

### Secondary analysis

The mean size of margins harvested by CO_2_ laser was 2.45 mm (95% CI 2.17-2.73 mm) and the mean size of margins harvested with steel instruments was 2.64 mm (95% CI 2.34-2.94 mm). This difference was not statistically significant (p = 0.37). The potential for laser to cause artifact by superficial vaporization of surface epithelium was also assessed (Table 4). After excluding deep margins, there were 89 margins taken by steel instruments and 94 margins taken by laser that were harvested from epithelial surfaces. Of these specimens, 36% and 38% of the margins harvested by steel and laser respectively had complete loss or destruction of the epithelium. Using Fisher’s exact test, the difference between these groups was not significant (p = 0.76). It is important to note that since the oncologic resection was performed by laser, at least one side of these already small margins would contain pre-existing thermal damage prior to margin harvest.

**Table 4:** Surface epithelium status of margin specimens based on technique

**Technique**	**Surface epithelium**		**Total**	**p-value**
	**Preserved**	**Complete loss**		
***Steel***	57 (64.0%)	32 (36.0%)	89 (100.0%)	p = 0.76
***Laser***	58 (61.7%)	36 (38.3%)	94 (100.0%)	

The planned logistic regression model for the relationship between technique and uninterpretable margins controlling for margin size could not be performed as all cases of uninterpretable margins occurred with steel instrument technique. Controlling for margin size, the laser technique for obtaining margins was associated with an odds ratio of 2.05 (95% CI 1.12-3.77) for obtaining either marked artifact or uninterpretable margins when compared to steel instrument technique. This relationship was statistically significant (p = 0.020) (Table 5). Independent of technique, an increase in margin size by 1 mm was associated with a non-significant (p = 0.31) decreased odds ratio of 0.9 for obtaining margins that were uninterpretable or had marked artifact.

**Table 5:** Logistic regression of relationship between excision technique and margins described as either marked artifact or uninterpretable while controlling for size

**Variable**	**Odds ratio**	**95% CI**	**p-value**
**Technique**			
*Laser vs. Steel*	2.05	1.12-3.77	0.020
**Size**			
Increase by 1 mm	0.90	0.74-1.10	0.31

## Discussion

Positive margin status in glottic cancer is associated with increased risk of local recurrence and decreased disease specific survival [11-14]. Given these associations, surgical re-excision is warranted whenever possible. Rates of local recurrence are also significantly higher for glottic cancers resected with margins containing severe dysplasia or carcinoma in situ. It is generally recommended that these cases be considered for further surgical management in order to appropriately clear the margin [39-41].

We have introduced a classification system for artifact assessment in laser surgery for upper aero-digestive tract tumors. In this study, degree of artifact was classified into four categories: none, minor, marked, and uninterpretable. The classification schema is based on the presence of artifact and, when artifact is present, takes into account the impact on patient management of the resulting ability, or lack thereof, to interpret presence of dysplasia and invasive disease.

All surgical instruments can cause histological artifacts on microscopic examination [27,42]. By causing tissue destruction or simulating pathological findings, the degree of artifact can affect the interpretability of surgical specimens. [43,44] In cases with artifact affecting the surface epithelium, the changes can either prevent the diagnosis of dysplasia or can simulate dysplasia in non-dysplastic tissue. [44] Therefore, the pathologist has to disregard the areas with artifact and assess only those areas without artifact if they exist. Additionally, the laser may cause higher incidence of complete or partial denudation of the surface epithelium, leaving only the lamina propria. The latter can be assessed for the presence or absence of invasion, but not dysplasia.

There have been no previous studies comparing the effect of the use of CO_2_ laser to steel instruments on the interpretation of glottic cancer margin specimens. Our results demonstrated that the use of CO_2_ laser for margin harvest is associated with significantly higher odds of obtaining either a marked artifact or uninterpretable margins (p = 0.024).

A surprising finding in our study was the higher proportion of uninterpretable margins, but lower proportion of margins with marked artifact in the steel instrument group. It was expected that the degree of artifact would be distributed along a continuum, and that a higher proportion of uninterpretable margins would accompany a higher proportion of margins with marked artifact. The isolated finding of higher rates of uninterpretability in the steel instrument group was not statistically significant (p = 0.24) and the disparity between groups may represent an inadequate sample size. Alternatively, it could be due to differing mechanisms of artifact generation given the potential for the laser to denude the surface epithelium of these small specimens. This makes assessment of dysplasia impossible, while preserving the ability to interpret invasive malignancy. Steel instruments may preserve the epithelium more frequently, but may be more prone causing crush injury to an entire specimen making it completely uninterpretable. These potential differences in the mechanisms of artifact generation and rates of surface epithelium preservation between techniques are not supported by our current data.

## Conclusion

Accurate assessment of surgical margins is a key factor in proper management and the predictive risk of local recurrence. The present study demonstrates that both steel instruments and CO_2_ laser cause a significant degree of artifact that can interfere with accurate margin assessment in TLM for early glottic cancer. The use of laser to harvest margins in our study was not associated with increased crude rates of uninterpretability for malignancy but was associated with increasing artifact affecting the ability of the pathologist to assess for dysplasia.

## Competing interests

The authors declare that they have no competing interests.

## Authors’ contributions

FM: Ethics approval, patient enrollment, & manuscript. MR: Statistics and Data analysis. RH, SMT & JT: head & neck surgeons involved in patient selection and enrollment. TB: laryngologist involved in patient selection and enrollment. MB: pathologist. MH: involved in manuscript review and data analysis. All authors read and approved the final manuscript.
